# Risk prediction for central lymph node metastasis in isolated isthmic papillary thyroid carcinoma by nomogram: A retrospective study from 2010 to 2021

**DOI:** 10.3389/fendo.2022.1098204

**Published:** 2023-01-17

**Authors:** Yu Zhao, Wei Shi, Fang Dong, Xiuhua Wang, Chong Lu, Chunping Liu

**Affiliations:** ^1^ Department of Thyroid and Breast Surgery, Union Hospital, Tongji Medical College, Huazhong University of Science and Technology, Wuhan, China; ^2^ Department of Ultrasound Medicine, Union Hospital, Tongji Medical College, Huazhong University of Science and Technology, Wuhan, China

**Keywords:** central lymph node metastasis, isthmic papillary thyroid carcinoma, prediction, risk model, thyroid surgery

## Abstract

**Background:**

Isthmic papillary thyroid carcinoma (IPTC) is an aggressive thyroid cancer associated with a poor prognosis. Guidelines elaborating on the extent of surgery for IPTC are yet to be developed. This study aims to construct and validate a model to predict central lymph node metastasis (CLNM) in patients with IPTC, which could be used as a risk stratification tool to determine the best surgical approach for patients.

**Methods:**

Electronic medical records for patients diagnosed with isolated papillary thyroid carcinoma who underwent surgery at Union Hospital, Tongji Medical College, Huazhong University of Science and Technology, from January 2010 to December 2021 were reviewed. All patients who underwent thyroidectomy with central neck dissection (CND) for isolated IPTC were included. We conducted univariate and multivariate logistic regression analyses to assess risk factors for ipsilateral and contralateral CLNM and the number of CLNM in IPTC patients. Based on the analysis, the nomogram construction and internal validations were performed.

**Results:**

A total of 147 patients with isolated IPTC were included. The occurrence of CLNM was 53.7% in the patients. We identified three predictors of ipsilateral CLNM, including age, gender, and size. For contralateral CLNM, three identified predictors were age, gender, and capsular invasion. Predictors for the number of CLNM included age, gender, capsular invasion, tumor size, and chronic lymphocytic thyroiditis (CLT). The concordance index(C-index) of the models predicting ipsilateral CLNM, contralateral CLNM, 1-4 CLNM, and ≥5 CLNM was 0.779 (95%CI, 0.704, to 0.854), 0.779 (95%CI, 0.703 to 0.855), 0.724 (95%CI, 0.629 to 0.818), and 0.932 (95%CI, 0.884 to 0.980), respectively. The corresponding indices for the internal validation were 0.756 (95%CI, 0.753 to 0.758), 0.753 (95%CI, 0.750 to 0.756), 0.706 (95%CI, 0.702 to 0.708), and 0.920 (95%CI, 0.918 to 0.922). Receiver operating characteristic (ROC) curves, calibration, and decision curve analysis (DCA) results confirmed that the three nomograms could precisely predict CLNM in patients with isolated IPTC.

**Conclusion:**

We constructed predictive nomograms for CLNM in IPTC patients. A risk stratification scheme and corresponding surgical treatment recommendations were provided accordingly. Our predictive models can be used as a risk stratification tool to help clinicians make individualized surgical plans for their patients.

## 1 Introduction

Papillary thyroid carcinoma (PTC) is the most common type of differentiated thyroid cancer (DTC), which tends to metastasize to the cervical lymph nodes (1). It is reported that 30–80% of individuals with thyroid carcinoma experience lymph node metastasis, most commonly metastasizes to the central compartment lymph nodes (2). Studies have shown lymph node metastases display a predictive value for disease-free interval (DFI) of PTC patients (3), the American Thyroid Association (ATA) Management Guidelines report that lymph node metastasis in the central region is an independent risk factor for recurrent thyroid cancer (4). Therefore, lymph node metastasis is a critical factor to consider when determining the surgical strategy for PTC.

The isthmus is the central part of the thyroid gland. It is directly anterior to the trachea, covered by the strap muscles. According to reports, the incidence of isthmic thyroid cancer ranges from 2.5% to 9.2%. Compared to those on the lateral lobes, isthmic thyroid cancers are more likely to metastasize to lymph nodes (5). Other than that, several studies suggested that IPTC is a more aggressive thyroid cancer with a worse prognosis, multifocality, capsular invasion, and extrathyroidal extension (ETE) (6–11).

Some studies consider total thyroidectomy as a safe surgical approach for isthmic thyroid cancer (12, 13). However, for locally advanced or metastatic thyroid cancer, the surgical extent and surgical approach should be decided according to patients’ condition (14). On the other hand, several studies regard lobectomy with isthmusectomy or isthmusectomy alone as an alternative for selected low-risk patients, with advantages such as fewer surgical complications and better-preserved thyroid function (15, 16). Guidelines on the scope of surgery and the inclusion of prophylactic central neck dissection (CND) for IPTC are still lacking. Previous clinical reports on IPTC primarily focused on minimizing surgical complications, while the importance of assessing lymph node metastasis for surgical decision-making is often ignored. Considering its significant impact on patient prognosis, a practical tool to assess the risk of lymph node metastasis is in demand, according to which more appropriate surgical decisions can be made.

In this study, we screened risk factors for lymph node metastasis according to the clinical and pathological characteristics of patients with isolated IPTC. Then we developed nomograms to predict the risk of lymph node metastasis in these patients. We hope the method we constructed can help with surgical decision-making for IPTC patients.

## 2 Materials and methods

### 2.1 Patients

**Figure 1:**
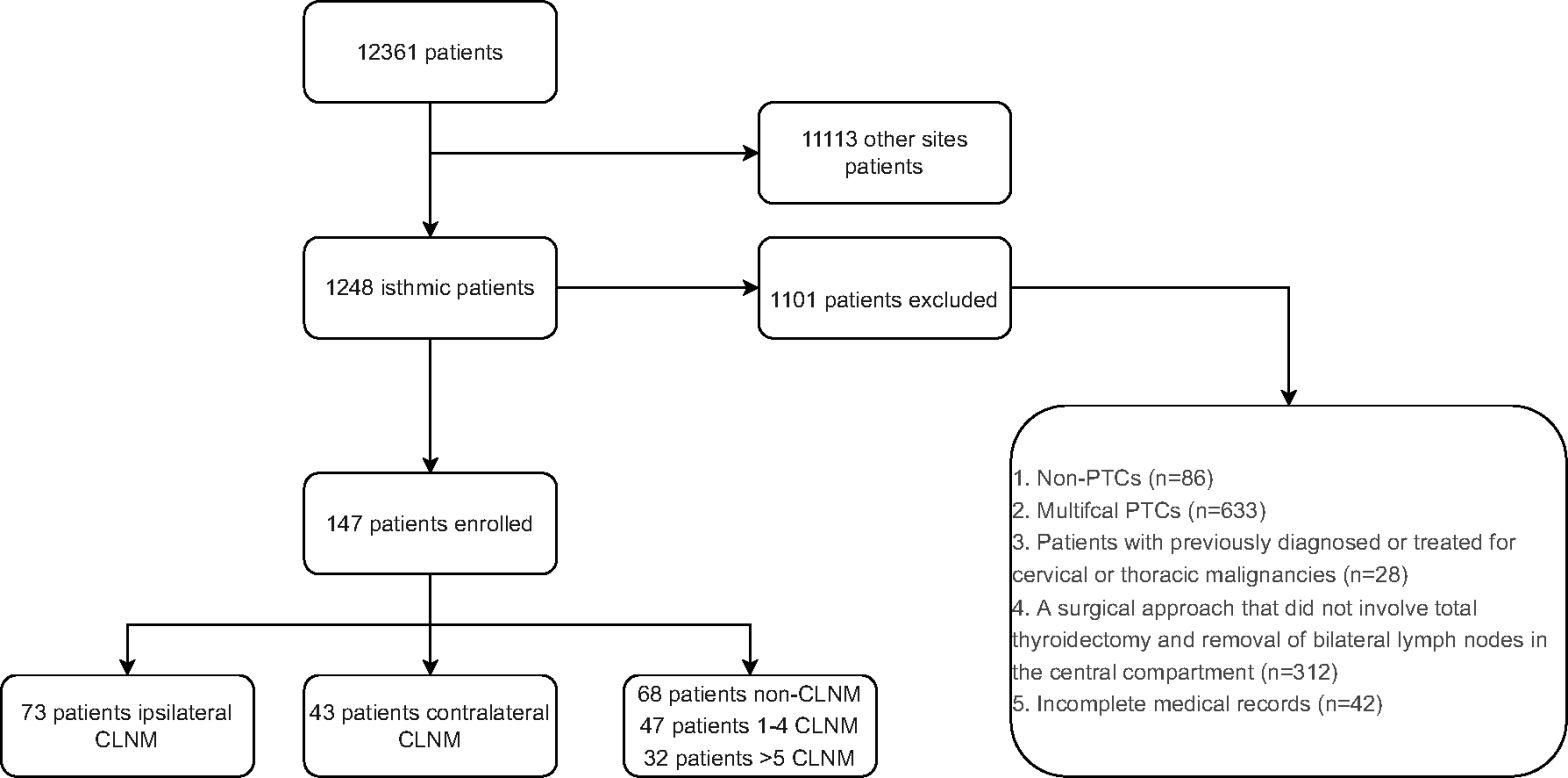
Flow chart of patient inclusion process. PTC, Papillary thyroid carcinoma; CLNM, central lymph node metastasis.

Electronic medical records for all adult patients with papillary thyroid cancer who underwent primary surgery from January 2010 to December 2021 in Union Hospital, Tongji Medical College, Huazhong University of Science and Technology were reviewed. Exclusion criteria were: (1) non-PTCs (medullary/follicular/anaplastic thyroid cancers), (2) multifocal PTCs, (3) previously diagnosed or treated cervical or thoracic malignancies, (4) surgical approach that did not involve total thyroidectomy and removal of bilateral lymph nodes in the central compartment, and (5) incomplete medical records. The flow chart exhibiting the patient selection process is shown in **Figure 1**.

All patients included in the study underwent bilateral thyroidectomy with CND. All central lymph nodes were removed, including prelaryngeal lymph nodes, pretracheal lymph nodes, and paratracheal lymph nodes, as the American Thyroid Association guidelines recommended. The surgeries were performed by a surgeon with at least ten years of experience in thyroid surgery. All surgical specimens were examined microscopically and cross-checked by two experienced pathologists.

### 2.2 Data collection

All data were retrieved from the Electronic Medical Record System by investigators. We collected data on demographic characteristics, serum thyroglobulin (Tg) and anti-Tg levels, thyroid ultrasonography (US) reports (including extracapsular relationships, shape, internal content, echogenicity, margins, and size), and the pathological findings of surgical specimens.

### 2.3 Definition

**Figure 2:**
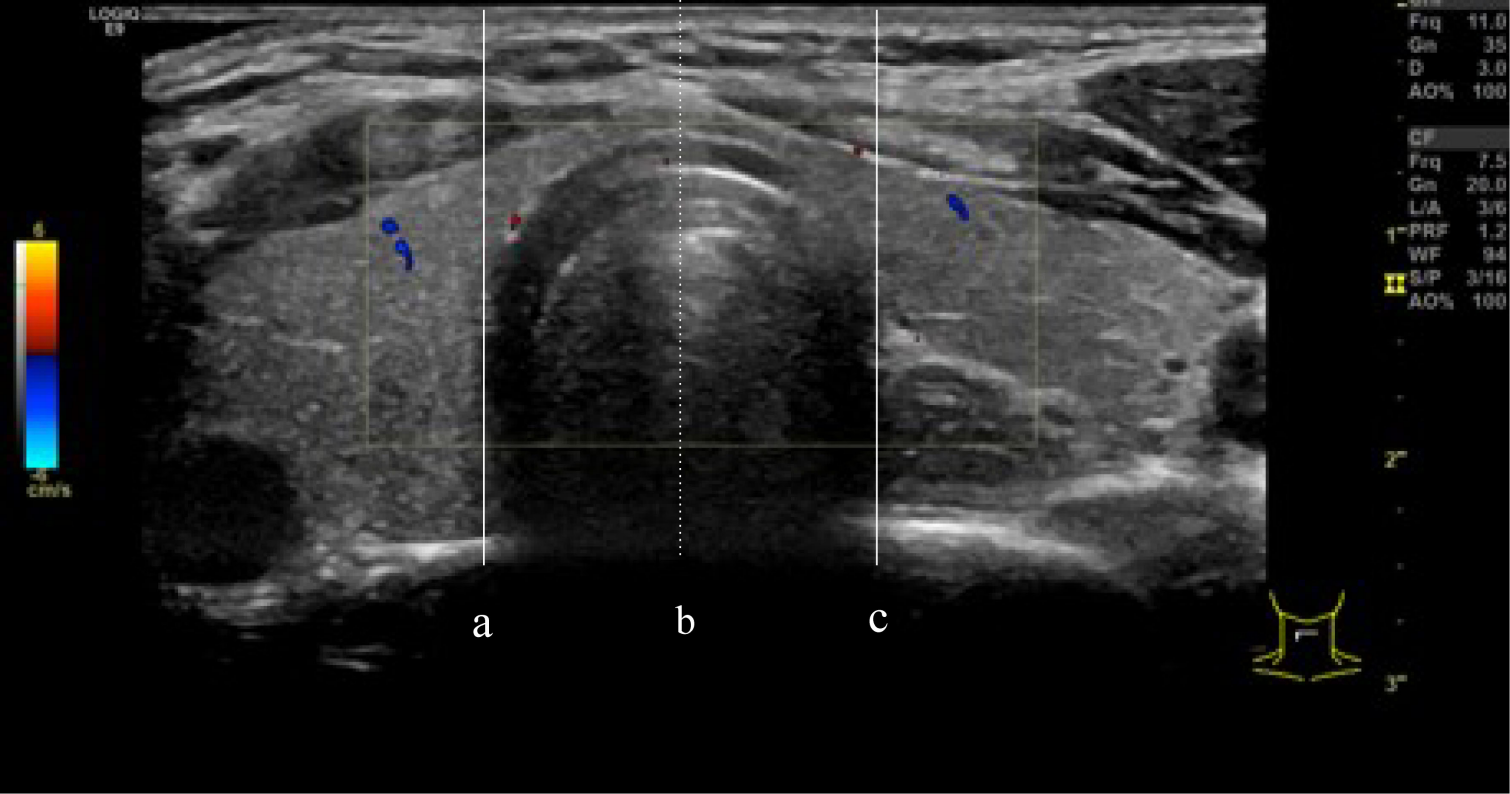
**(A, C)** are defined as the boundaries of the trachea, the isthmus thyroid was defined as the part of the thyroid gland between line **(A)** and line **(C)**. line **(B)** divided the isthmic thyroid into 2 equal parts. right isthmus and left isthmus.

This study defined isthmic thyroid nodules as those located in the thyroid tissue in front of the trachea, bordered by the projection of the trachea on the thyroid gland. The midline of the trachea subdivided the isthmic thyroid nodules into left and right isthmus groups. We made these position descriptions according to the location of the nodule center, defined as the intersection of the long and short axes of the nodule (17) (**Figure 2**).

The capsule invasion was determined either by pathology or by US finding of interruption of the capsule. The extrathyroidal extension was determined by pathology. Nodular goiter was defined as anechoic nodules on the US with benign pathology. Chronic lymphocytic thyroiditis was determined by elevated thyroid antibodies, elevated peroxidase level, and findings of diffuse heterogeneity on US or pathology. Central lymph node invasions were assessed by postoperative pathology.

### 2.4 Statistical analysis

All statistical analyses were performed using the SPSS version 24.0 software (Chicago, IL) and R software version 4.2.1 (The R Foundation for Statistical Computing). Continuous data were tested for normality and reported as the mean and standard deviation (SD). Differences between groups were analyzed using the independent t-test, Pearson Chi-square statistic, or Fisher exact test. Two-tailed P values of <0.05 were considered to indicate statistical significance. Variables with P < 0.05 in the univariate analyses were included in the multivariate logistic regression. Variables with P <0.05 in the multivariate analysis were then used to construct a risk prediction model. Nomogram was used to present the results. We assessed the performance of this model with ROC curves, calibration charts, and decision curves. All models were internally validated using the bootstrap resampling procedure.

## 3 Results

### 3.1 Patient basic characteristics

A total of 12,361 patients diagnosed with PTC who underwent thyroid surgery at Union Hospital Tongji Medical College, Huazhong University of Science and Technology were included. Among them, 1,248 patients had IPTC (10.09%). After excluding patients according to the criteria described above (see Methods), 147 patients were finally enrolled, and 1,101 patients were excluded (**Figure 1**). These 147 patients included 107 women and 40 men with a mean age of 41.93 years (range 15-71 years). Among 147 patients, 73 patients (49.67%) had ipsilateral CLNM, and 43 (29.3%) had contralateral CLNM. 68 (46.3%) patients had no lymph node metastasis, 47 (32%) had 1-4 CLNM, and 32 (21.7%) had more than four CLNM. Patients’ baseline data are shown in **Tables 1**, **2**.

**Table 1:** Baseline characteristics of patients in isthmus-originating papillary thyroid carcinoma (PTC).

	Ipsilateral CLNM		Contralateral CLNM	
Characteristic	Absence (n=74)	Presence (n=73)	Absence (n=104)	Presence (n=43)
Gender, n (%)				
Male	13 (17.6%)	27 (37%)	23 (22.1%)	17 (39.5%)
Female	61 (82.4%)	46 (63%)	81 (77.9%)	26 (60.5%)
Age (years)				
Mean ± SD [range]	44.39 ± 10.4	39.42 ± 11.088	43.82 ± 10.462	37.35 ± 11.028
nodular goiter, n (%)				
Presence	48 (64.9%)	35 (47.9%)	62 (59.6%)	21 (48.8%)
Absence	26 (35.1%)	38 (52.1%)	42 (40.4%)	22 (51.2%)
CLT, n (%)				
Presence	33 (44.6%)	18 (24.7%)	40 (38.5%)	31 (72.1%)
Absence	41 (55.4%)	55 (75.3%)	64 (61.5%)	12 (27.9%)
Size (cm)				
Mean ± SD (range)	9.12 ± 4.97	16.55 ± 11.89	12.07 ± 9,5	14.61 ± 10.34
≤1cm	51 (68.9%)	22 (30.1%)	59 (56.7%)	14 (32.6%)
≤2cm	19 (25.7%)	30 (41.1%)	28 (26.9%)	21 (48.8%)
>2cm	4 (5.4%)	21 (28.8%)	17 (16.3%)	8 (18.6%)
Composition,n (%)				
Solid	73 (98.6%)	67 (91.8%)	98 (94.2%)	42 (97.7%)
non-Solid	1 (1.4%)	6 (8.2%)	6 (5.8%)	1 (2.3%)
Echogenic foci,n (%)				
Presence	50 (67.6%)	64 (87.7%)	77 (74%)	37 (86%)
Absence	24 (32.4%)	9 (12.3%)	27 (26%)	6 (14%)
Echogenicity,n (%)				
Hypoechoic	71 (95.9%)	64 (87.7%)	97 (93.3%)	38 (88.4%)
Hyper- or equal-echogenicity	3 (4.1%)	9 (12.3%)	7 (6.7%)	5 (11.6%)
Shape,n (%)				
Taller than wide	16 (21.6%)	10 (13.7%)	20 (19.2%)	6 (14%)
Non-ovoid	58 (78.4%)	63 (86.3%)	84 (80.8%)	37 (86%)
Margin,n (%)				
Regular	15 (20.3%)	9 (12.3%)	21 (20.2%)	3 (7%)
Irregular	59 (79.7%)	64 (87.7%)	83 (79.2%)	40 (79.8%)
Capsule,n (%)				
None-capsule invasion	25 (33.8%)	14 (19.2%)	33 (31.7%)	6 (14%)
Capsular invasion	42 (56.8%)	41 (56.2%)	59 (56.7%)	24 (55.8%)
ETE	7 (9.4%)	18 (20.5%)	12 (11.6%)	13 (30.2%)

Continuous variables are reported as mean (standard deviation); categorical variables, as frequencies (proportions). PTC, papillary thyroid carcinoma; CLNM, central lymph node metastasis; SD, standard deviation;CLT, chronic lymphocytic thyroiditis; ETE, extrathyroidal extension.

**Table 2:** Baseline characteristics of patients in isthmus-originating papillary thyroid carcinoma (PTC).

	Number Of CLNM		
Characteristic	Non (n=68)	1-4 (n=47)	>5 (n=32)
Gender, n (%)			
Male	10 (14.7%)	13 (27.7%)	17 (15.3%)
Female	58 (85.3%)	34 (72.3%)	15 (46.9%)
Age (years)			
Mean ± SD [range]	44.85 ± 10.527	42.53 ± 9.371	34.81 ± 11.318
nodular goiter, n (%)			
Presence	44 (64.7%)	25 (53.2%)	14 (43.8%)
Absence	24 (35.3%)	22 (46.8%)	18 (56.3%)
CLT, n (%)			
Presence	31 (45.6%)	16 (34%)	4 (12.5%)
Absence	37 (54.4%)	31 (66%)	28 (87.5%)
Size (cm)			
Mean ± SD (range)	9.33 ± 5.67	14.34 ± 11.32	17.95 ± 11.59
≤1cm	47 (69.1%)	21 (44.7%)	5 (15.6%)
≤2cm	17 (25%)	14 (29.8%)	18 (56.3%)
>2cm	4 (5.9%)	12 (25.5%)	9 (28.1%)
Composition,n (%)			
Solid	67 (98.5%)	42 (89.4%)	31 (96.9%)
non-Solid	1 (1.5%)	5 (10.6%)	1 (3.1%)
Echogenic foci,n (%)			
Presence	46 (67.6%)	38 (80.9%)	30 (93.8%)
Absence	22 (32.4%)	9 (19.1%)	2 (6.3%)
Echogenicity,n (%)			
Hypoechoic	15 (22.1%)	7 (14.9%)	4 (12.5%)
Hyper- or equal-echogenicity	53 (77.9%)	40 (85.1%)	28 (87.5%)
Shape,n (%)			
Taller than wide	15 (22.1%)	7 (14.9%)	4 (12.5%)
Non-ovoid	53 (77.9%)	40 (85.1%)	28 (87.5%)
Margin,n (%)			
Regular	14 (20.6%)	10 (21.3%)	0 (0)
Irregular	54 (79.4%)	37 (78.7%)	32 (100%)
Capsule,n (%)			
None-capsule invasion	25 (36.8%)	11 (23.4%)	3 (9.4%)
Capsular invasion	36 (52.9%)	28 (59.6%)	19 (59.4%)
ETE	7 (10.3%)	8 (17.0%)	10 (31.3%)

Continuous variables are reported as mean (standard deviation); categorical variables, as frequencies (proportions). PTC, papillary thyroid carcinoma; CLNM, central lymph node metastasis; SD, standard deviation;CLT, chronic lymphocytic thyroiditis; ETE, extrathyroidal extension.

### 3.2 Predictors for ipsilateral CLNM

Univariable and multivariable logistic regression was performed to assess variables associated with ipsilateral CLNM (**Table 3**). In the univariate analysis, gender (male: OR= 2.754, 95%CI, 1.282 to 5.915), tumor size (OR=1.134, 95%CI, 1.070 to 1.203), age (OR=0.958, 95%CI, 0.928 to 0.988), echogenic foci (OR=3.413, 95%CI, 1.458 to 7.992), nodular goiter (OR=0.499, 95%CI, 0.257 to 0.967), CLT (OR=0.407, 95%CI, 0.201 to 0.821), and capsule (capsular invasion: OR=1.743, 95%CI, 0.797 to 3.814; ETE: OR=4.952, 95%CI, 1.542 to 13.671) were associated with ipsilateral CLNM(P<0.05). In the multivariate analysis of the seven variables, gender, age, and tumor size were significantly related to ipsilateral CLNM (P<0.05). Features including larger tumor size (OR=1.135, 95%CI,1.067 to 1.207) and being male (OR=2.448, 95%CI,1.037 to 5.779) were associated with a higher risk of ipsilateral CLNM. Older age (OR=0.962,95%CI, 0.926 to 0.999) was associated with a lower risk of ipsilateral CLNM.

**Table 3:** Univariate analysis and multivariate analysis of factors associated with ipsilateral central lymph node metastasis (CLNM) in patients with isthmus papillary thyroid carcinoma (PTC).

Characteristic	Univariate analysis			Multivariate analysis		
	*P*	OR	OR (95%CI)	*P*	OR	OR (95%CI)
Gender, n(%)						
Male	0.009	2.754	1.282,5.915	0.041	2.448	1.037,5.779
Age(years)	0.007	0.958	0.928,0.988	0.043	0.962	0.926,0.999
nodular goiter, n(%)						
Presence	0.040	0.499	0.257,0.967	0.051	0.445	0.197,1.004
CLT, n(%)						
Presence	0.012	0.407	0.201,0.821	0.056	0.455	0.203,1.021
Size (cm)						
Mean ± SD [range]	0	1.134	1.070,1.203	0	1.135	1.067,1.207
≤1cm						
≤2cm	0.001	3.660	1.709,7.840			
>2cm	0	12.170	3.738,39.621			
Composition,n(%)						
Solid	0.086	0.153	0.180,1.304			
Echogenic foci,n(%)						
Presence	0.005	3.413	1.458,7.992	0.225	1.834	0.688,4.890
Echogenicity,n(%)						
Hypoechoic	0.081	0.300	0.780,0.159			
Shape,n(%)						
Taller than wide	0.211	0.575	0.242,1.369			
Margin,n(%)						
Irregular	0.197	1.808	0.736,4.442			
Capsule,n(%)						
None-capsule invasion				0.616		
Capsular invasion	0.164	1.743	0.797,3.814	0.521	1.358	0.534,3.453
ETE	0.006	4.952	1.542,13.671	0.335	1.914	0.512,7.152

CLNM, central lymph node metastasis; PTC, papillary thyroid carcinoma; OR, odds Ratio; CI confidence interval; CLT, chronic lymphocytic thyroiditis; SD, standard deviation; ETE, extrathyroidal extension.

### 3.3 Predictors for contralateral CLNM

Univariable and multivariable logistic regression was performed to assess variables associated with contralateral CLNM (**Table 4**). In the univariate analysis, age (OR=0.945,95%CI, 0.913 to 0.979), gender (male: OR=2.303,95%CI, 1.069 to 4.958), tumor size (OR=1.025, 95%CI, 0.990 to 1.061), and capsule condition (capsular invasion: OR=2.237, 95%CI, 0.831 to 6.026; ETE: OR=5.958, 95%CI, 1.847 to 19.225) were associated with contralateral CLNM (P<0.05). In the multivariate analysis, capsular invasion and being male was associated with a higher risk of contralateral CLNM (capsular invasion: OR=2.060, 95%CI, 0.711 to 5.966; ETE: OR=8.268, 95%CI, 2.263 to 30.265; male: OR=2.430, 95%CI, 1.046 to 5.648), and the older age was associated with lower risk of contralateral CLNM. (OR=0.929, 95%CI, 0.892 to 0.968).

**Table 4:** Univariate analysis and multivariate analysis of factors associated with contralateral central lymph node metastasis (CLNM) in patients with isthmus papillary thyroid carcinoma (PTC).

Characteristic	Univariate analysis			Multivariate analysis		
	*P*	OR	OR (95%CI)	*P*	OR	OR (95%CI)
Gender, n(%)						
Male	0.033	2.303	1.069,4.958	0.039	2.430	1.046,5.648
Age(years)	0.002	0.945	0.913,0.979	0	0.929	0.892,0.968
nodular goiter, n(%)						
Presence	0.232	0.647	0.316,1.322			
CLT, n(%)						
Presence	0.138	0.550	0.249,1.213			
Size (cm)	0.162	1.025	0.990,1.061	0.750	0.993	0.951,1.037
≤1cm	0.210					
≤2cm	0.005	3.161	1.403,7.120			
>2cm	0.189	1.983	0.713,5.514			
Composition,n(%)						
Solid	0.389	2.571	0.300,22.023			
Echogenic foci,n(%)						
Presence	0.188	2.162	0.822,5.690			
Echogenicity,n(%)						
Hypoechoic	0.330	0.548	0.164,1.834			
Shape,n(%)						
Taller than wide	0.447	0.681	0.253,1.835			
Margin,n(%)						
Irregular	0.060	3.373	0.950,11.979			
Capsule,n(%)						
None-capsule invasion	0.024			0.006		
Capsular invasion	0.111	2.237	0.831,6.026	0.183	2.060	0.711,5.966
ETE	0.003	5.958	1.847,19.225	0.001	8.268	2.263,30.265

CLNM, central lymph node metastasis; PTC, papillary thyroid carcinoma; OR, odds Ratio; CI confidence interval; CLT, chronic lymphocytic thyroiditis; SD, standard deviation; ETE, extrathyroidal extension.

### 3.4 Predictors for the number of CLNM

Similarly, we performed univariable and multivariable analyses to assess variables related to the number of CLNM (**Table 5**). In the univariate analysis, gender(male: OR=4.007,95%CI, 1.984 to 8.092), age (OR=0.940,95%CI, 0.912 to 0.969), nodular goiter (OR=0.527, 95%CI, 0.285 to 0.975), CLT (OR=0.353,95%CI, 0.180 to 0.693), tumor size (OR=1.074, 95%CI, 1.035 to 1.115), echogenic foci (OR=3.220,95%CI, 1.444 to 7.181), and capsule condition (capsular invasion: OR=2.478, 95%CI, 1.154 to5.321; ETE: OR=5.330, 95%CI, 1.988 to14.295) were related to the number of CLNM (P<0.05). In the multivariate analysis, gender, age, tumor size, CLT, and capsule were associated with the number of CLNM(P<0.05). Capsule, male, and larger tumor size were associated with a higher risk of the number of CLNM (capsular invasion: OR=2.207, 95%CI: 0.930 to5.241; ETE: OR=4.168,95%CI, 1.339 to 12.979; male: OR=3.013,95%CI, 1.403 to 6.469, size: OR=1.052,95%CI,1.011 to1.094). The older age and CLT were associated with a lower risk of the number of CLNM. (age: OR=0.937,95%CI, 0.094 to 0.971, CLT: OR=0.385, 95%CI, 0.182 to 0.816).

**Table 5:** Univariate analysis and multivariate analysis of factors associated with number of central lymph node metastasis (CLNM) in patients with isthmus papillary thyroid carcinoma (PTC).

Characteristic	Univariate analysis			Multivariate analysis		
	*P*	OR	OR (95%CI)	*P*	OR	OR (95%CI)
Gender, n(%)						
Male	0	4.007	1.984,8.092	0.005	3.013	1.403,6.469
Age(years)	0	0.940	0.912,0.969	0	0.937	0.904,0.971
nodular goiter, n(%)						
Presence	0.041	0.527	0.285,0.975	0.316	0.694	0.340,1.417
CLT, n(%)						
Presence	0.002	0.353	0.180,0.693	0.013	0.385	0.182,0.816
Size(cm)	0	1.074	1.035,1.115	0.013	1.052	1.011,1.094
≤1cm						
≤2cm	0	7.897	2.687,23.207			
>2cm	0.001	7.650	2.255,25.947			
Composition,n(%)						
Solid	0.398	0.547	0.135,2.213			
Echogenic foci,n(%)						
Presence	0.004	3.220	1.444,7.181	0.170	1.900	0.759,4.756
Echogenicity,n(%)						
Hypoechoic	0.064	0.353	0.117,1.064			
Shape,n(%)						
Taller than wide	0.388	0.578	0.254,1.317			
Margin,n(%)						
Irregular	0.057	2.343	0.973,5.639			
Capsule,n(%)						
None-capsule invasion						
Capsular invasion	0.020	2.478	1.154,5.321	0.073	2.207	0.930,5.241
ETE	0.001	5.330	1.988,14.295	0.014	4.168	1.339,12.979

CLNM, central lymph node metastasis; PTC, papillary thyroid carcinoma; OR, odds Ratio; CI confidence interval; CLT, chronic lymphocytic thyroiditis; SD, standard deviation; ETE, extrathyroidal extension.

### 3.5 Development and validation of prediction models

**Figure 3:**
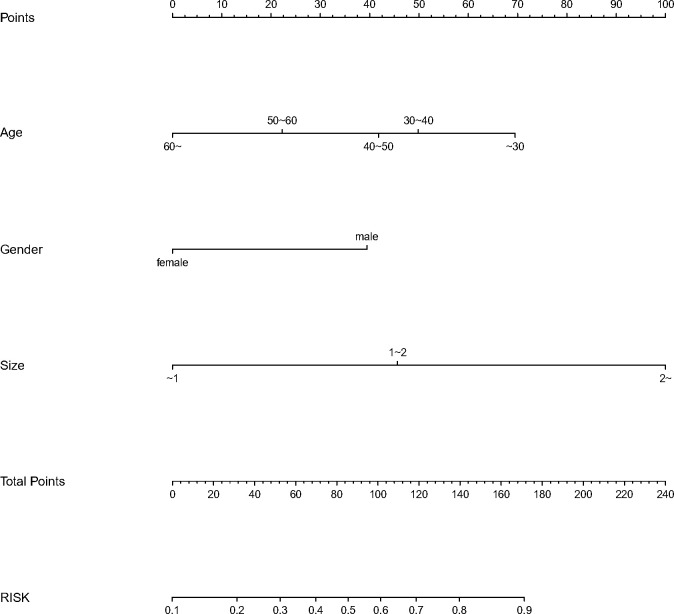
Nomogram to predict the risk of ipsilateral (CLNM)for patients with (IPTC). CLNM, central lymph node metastasis; IPTC, isthmic papillary thyroid carcinoma.

**Figure 4:**
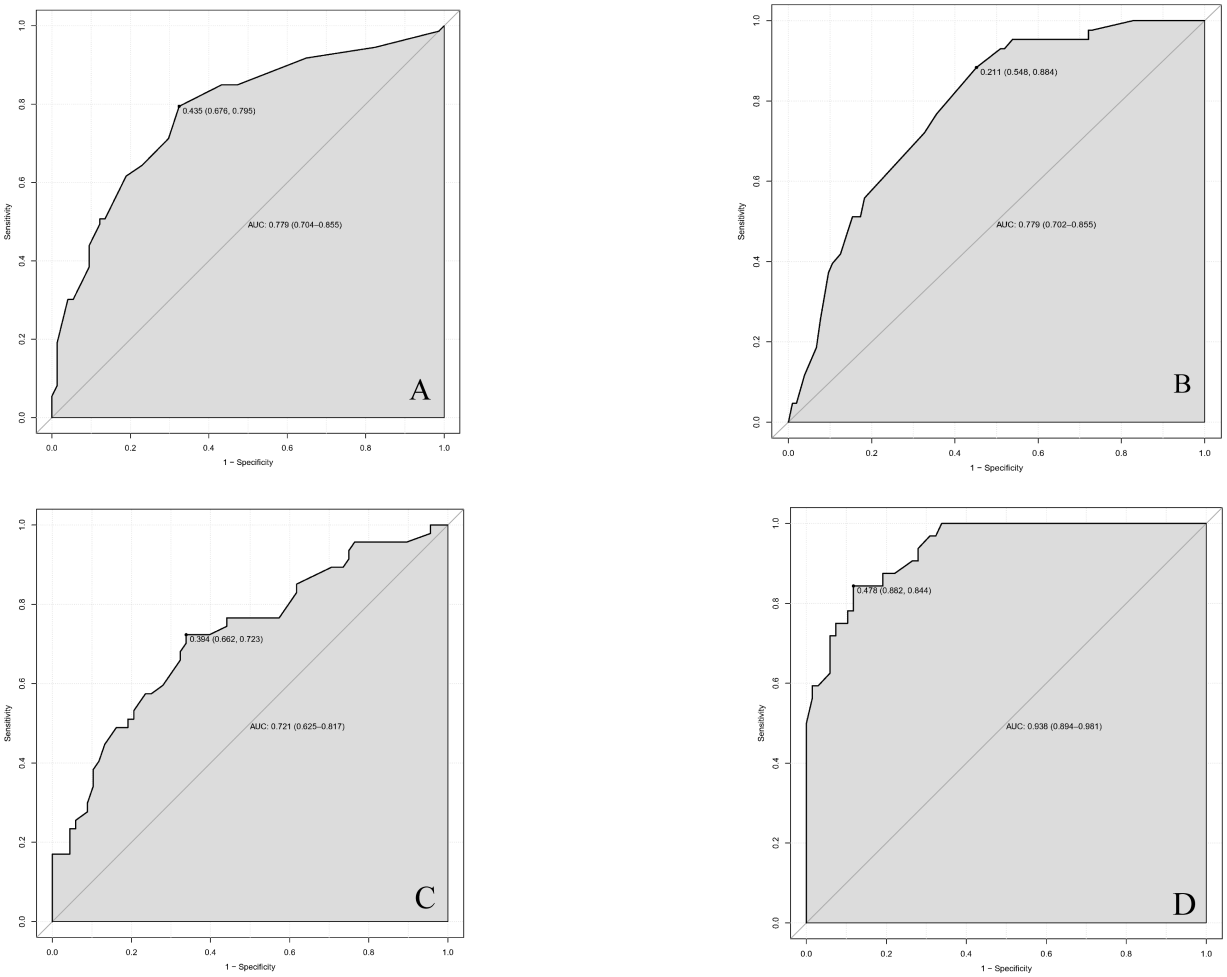
Receiver operating characteristic (ROC) curves in **(A)** ipsilateral central lymph node metastasis (CLNM), **(B)** contralateral central lymph node metastasis (CLNM), **(C)** 1-4 central lymph node metastasis (CLNM), **(D)** >5 central lymph node metastasis (CLNM) for nomogram models. AUC(A)=0.779 (95%CI,0.704-0.855); AUC(B)=0.779 (95%CI,0.702-0.855); AUC(C)=0.721 (95%CI,0.625-0.817); AUC(D)=0.938 (95CI%,0.894-0.981) ROC, receiver operating characteristic curves; AUC, area under the curve. CLNM, central lymph node metastasis. CI, confidence interval.

**Figure 5:**
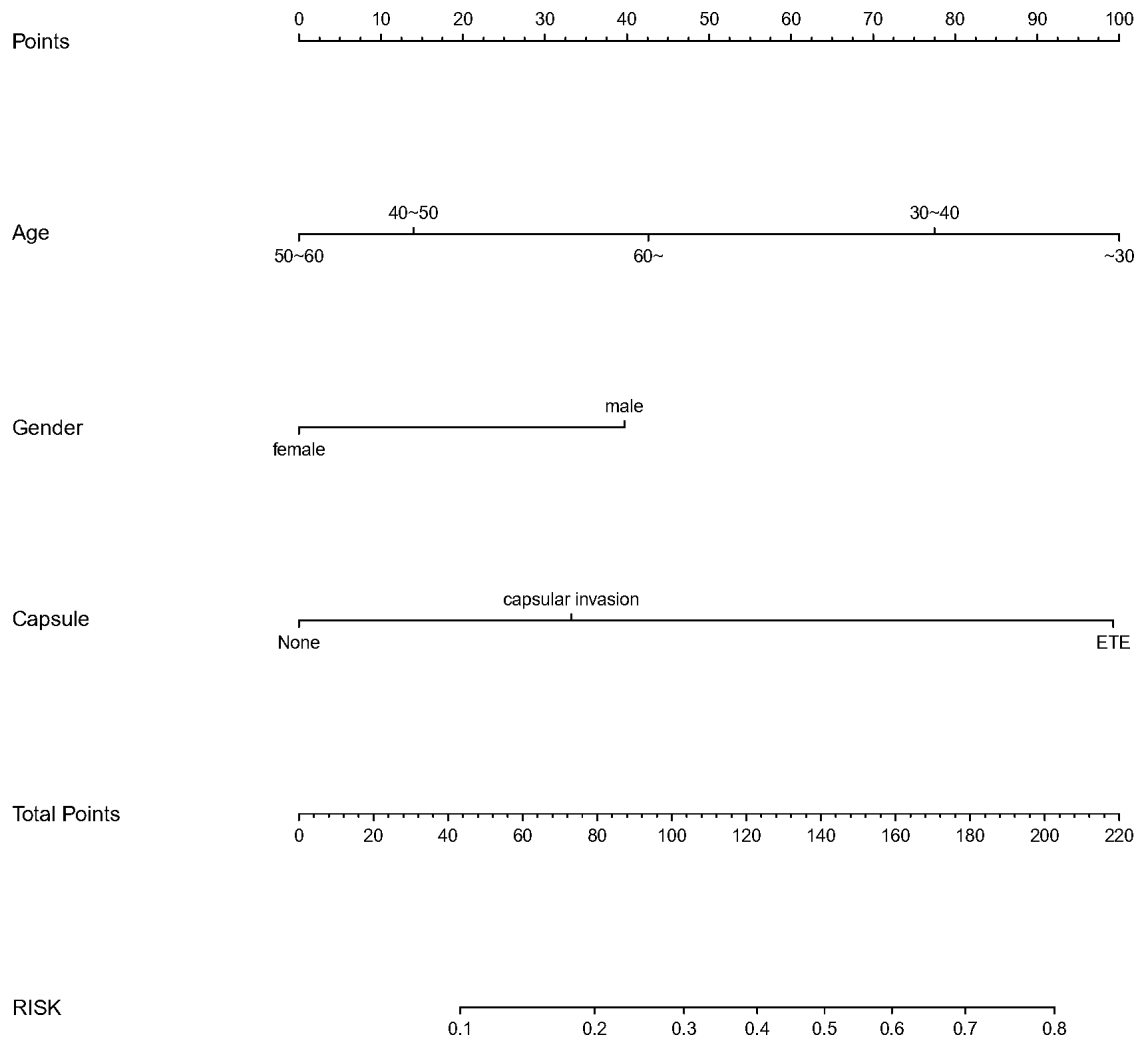
Nomogram to predict the risk of contralateral central lymph node metastasis(CLNM)for patients with isthmic papillary thyroid carcinoma (IPTC).CLNM,centrallymphnodemeta stasis; IPTC,isthmicpapillarythyroidcarcinom a.

**Figure 6:**
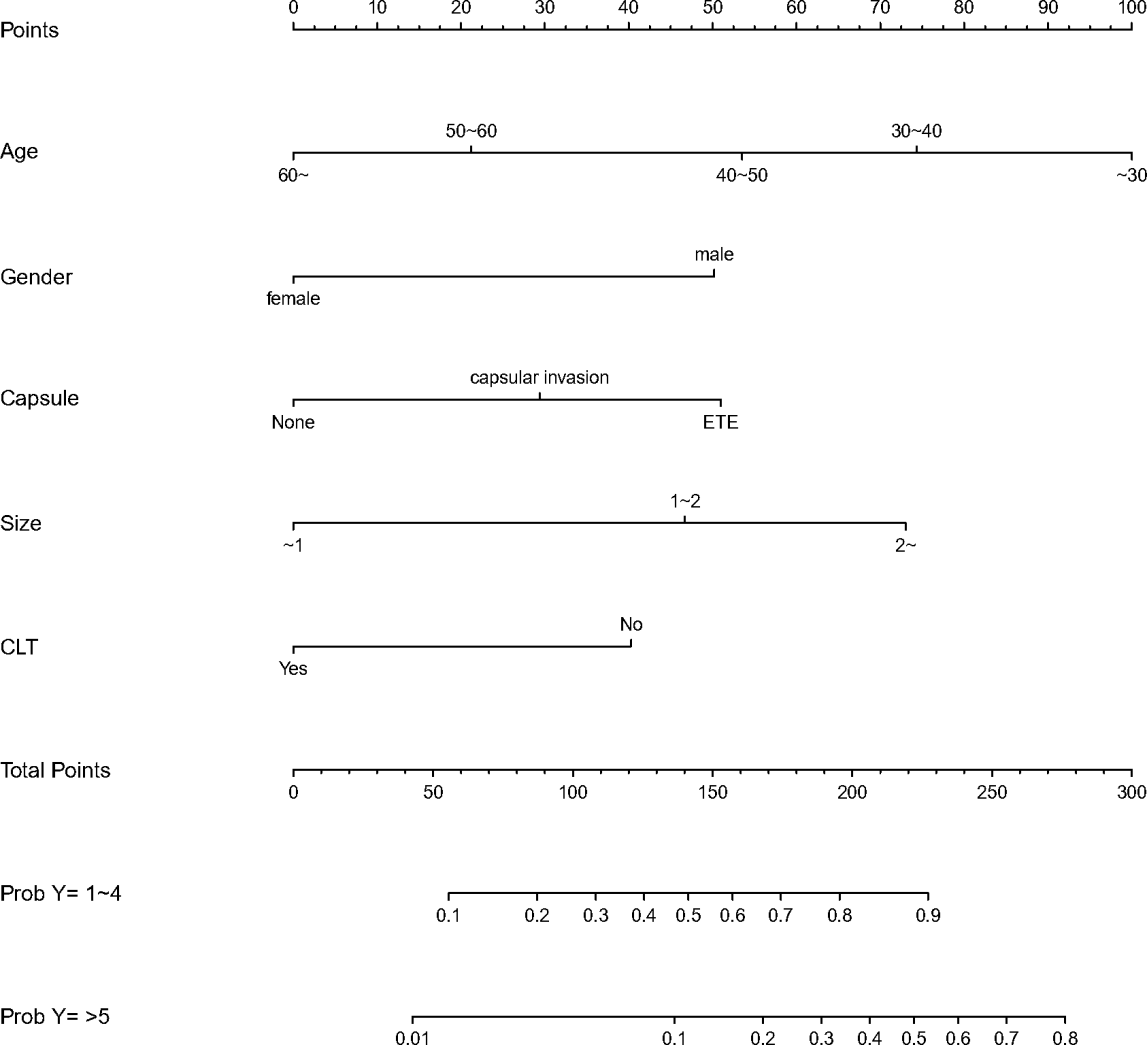
Nomogram to predict the central lymph node metastasis(CLNM) number for patients with isthmic papillary thyroid carcinoma(IPTC). CLNM,central lymph node metastasis; IPTC,isthmic papillary thyroid carcinoma.

**Figure 7:**
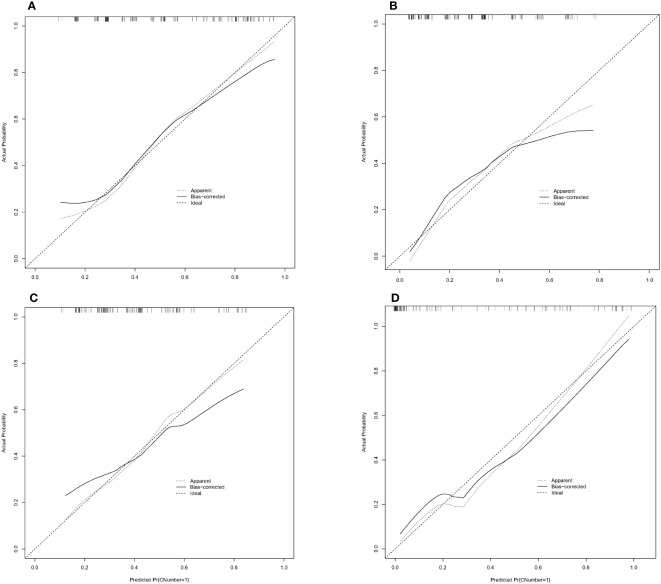
The calibration curve for **(A)** ipsilateral central lymph node metastasis (CLNM), **(B)** contralateral central lymph node metastasis (CLNM), **(C)** 1-4 central lymph node metastasis (CLNM), **(D)** >5 central lymph node metastasis (CLNM).

**Figure 8:**
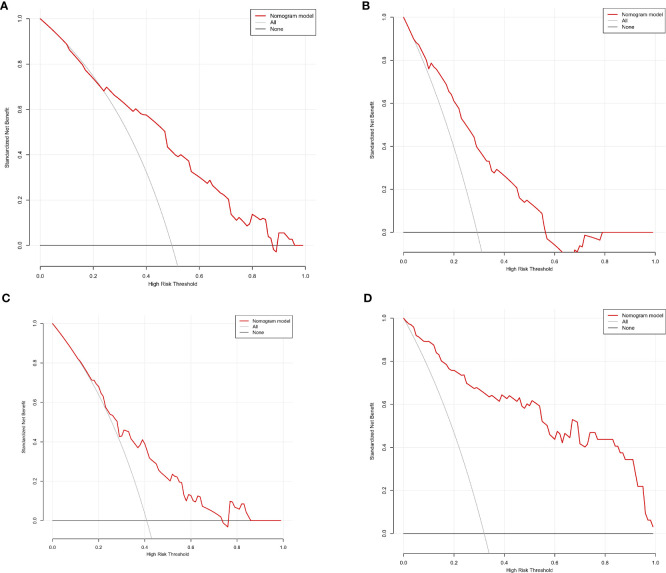
Decision curve analysis (DCA) for **(A)** ipsilateral central lymph node metastasis (CLNM), **(B)** contralateral central lymph node metastasis (CLNM), **(C)** 1-4 central lymph node metastasis (CLNM), **(D)** >5 central lymph node metastasis (CLNM).

We finally integrated the above predictors into our risk prediction models. The results of the variance inflation factor showed that one of these variables had covariance. We used three predictors in the nomogram to predict the risk of ipsilateral CLNM for patients with IPTC (**Figure 3**). The area under the ROC curve (AUROC) of the model was 0.779 (95%CI, 0.704 to 0.855) (**Figure 4A**). Three predictors were included to construct the nomogram for contralateral CLNM prediction (**Figure 5**). The AUROC of the model was 0.779 (95%CI, 0.702 to 0.855) (**Figure 4B**). Similarly, five predictors were included in the nomogram for CLNM number prediction (**Figure 6**). The AUROC of the model for predicting 1-4 CLNM was 0.721 (95%CI, 0.625 to 0.817) (**Figure 4C**), and that for predicting >5 CLNM was 0.938 (95%CI, 0.894 to 0.981) (**Figure 4D**). Furthermore, we validated the model internally by bootstrapping with 1000 resamples and conducting the calibration plot for the established models. In models for predicting ipsilateral and contralateral CLNM, C-indexes were 0.756 (95%CI, 0.753 to 0.758) and 0.753 (95%CI, 0.750 to 0.756), respectively. In models for predicting 1~4 CLNM and>5 CLNM, C-indexes were 0.706 (95%CI, 0.702 to 0.708) and 0.920 (95%CI, 0.918 to 0.922). The calibration curves of all prediction models are shown in **Figure 7**. All the calibration curves showed good linearity. To determine the clinical usefulness of the models, we quantified the net benefits at different threshold probabilities. In patients with IPTC, when the ipsilateral CLNM threshold probability of a patient was25%-87%, clinical decision guided by the prediction model would provide more benefit than either the treat-all-patient scheme or the treat-no-patient scheme (**Figure 8A**). The corresponding threshold probabilities were 5%-56% for contralateral CLNM (**Figure 8B**), 17%-86% for 1-4 CLNM (**Figure 8C**), and 1%-99% for >5 CLNM (**Figure 8D**).

### 3.6 Risk stratification and the corresponding score of the predictive nomogram

Patients were reordered according to the total score of nomograms and divided into three equal groups: low-risk group, moderate-risk group, and high-risk group (**Table 6**). Each group’s risk for ipsilateral CLNM was approximately 30%, 30~60%, and 80%, respectively. Their risks for contralateral CLNM were around 20%, 20~35%, and 35%. The risks of 1-4 CLNM in low-, moderate-, and high-risk groups were about 40%, 40~80%, and 80%, respectively, and the risks of >5 CLNM were about 10%, 10-30%, and 30%.

**Table 6:** Metastasis risk stratification of patients with papillary thyroid carcinoma (PTC) based on proportion of cohort.

Nomogram	Ipsilateral CLNM			Contralateral CLNM			Number of CLNM			
	Proportion	Score	Risk	Proportion	Score	Risk	Proportion	Score	Risk of 1~4	Risk of >5
Low risk	34%	<50	<30%	34	<54	<20%	34%	123	<40%	<10%
Moderated risk	34%	50~96	30%~60%	36.7	54~110	20~35%	34%	123~187	40~80%	10~30%
High risk	32%	>96	>60%	29.30%	>110	>35%	32%	>187	>80%	>30%

PTC, papillary thyroid carcinoma; CLNM, central lymph node metastasis.

## 4 Discussion

In this study, we established and validated prediction models for ipsilateral CLNM, contralateral CLNM, and the number of CLNM in unifocal IPTC. Based on the score of nomograms, we proposed a risk stratification of lymph node metastasis, and we suggested that surgical treatment plans should be developed based on different risk scores. Our model enables individualized treatment strategies with higher effectiveness and lower surgical risks.

The presence and number of CLNM are important in surgical decision-making (3). Currently, US and computed tomography (CT) are the main preoperative examination methods for lymph node metastasis. The diagnostic accuracy of CT and US for lymph node metastasis in PTC was limited. The sensitivity and specificity of US for lateral lymph node metastasis (LLNM) are 75.8% and 88.0%, respectively, while those for CLNM are 28.4% and 95.0%, respectively. Meanwhile, the dependence of the US’s diagnostic performance on operators’ experience makes it lacking comparability and repeatability. The sensitivity and specificity of CT for LLNM are 81.1% and 84.0%, respectively, while those for CLNM are 40.0% and 89.5%, respectively (18). Some research showed that contrast-enhanced ultrasound (CEUS) could identify the micro-vascularization of lymph nodes and focal thickening of the cortex, which enables the higher diagnostic accuracy of lymph node metastases in thyroid cancer when combined with traditional US. Nonetheless, no clear evidence showed that CEUS could improve the sensitivity of diagnosing CLNM (19). In addition, the broad application of artificial intelligence (AI) in medical diagnosis has revolutionized the assessment of thyroid nodules, effectively improving its accuracy and sensitivity. Unfortunately, no evidence has proved AI’s application in the assessment of CLNM (20). Besides, neither CT nor US can predict the number of CLNM.

Therefore, many researchers have committed to developing a model for predicting lymph node metastasis in PTC, which reveals tumor size, age, and extrathyroidal extension as the independent risk factors for lymph node metastasis (21, 22).。However, due to the more aggressive clinical features of IPTC than other PTCs (1, 5, 12, 23), the applicability of those models in IPTC is uncertain. A previous model by Feng JW et al. (17) only predicted the location of lymph node metastasis and included mainly postoperative pathologies, lacking clinical significance in surgical approach selection. In the meanwhile, some studies showed that larger thyroid volume and higher BMI could add to the surgical difficulty and prolong surgical duration (24). Therefore, for these patients, a better approach to assessing surgical extent is needed. Thus, it is urgent to develop a CLNM prediction model for IPTC patients and provide evidence for surgical decision-making is urgent.

Here we not only identified the risk factors of ipsilateral and contralateral CLNM but also grouped the number of CLNMs according to the risk stratification of the 2015 ATA guidelines (4). Moreover, factors associated with the risk stratification of the number of CLNM were identified. We found that age, gender, and tumor size can be used to predict ipsilateral CLNM; capsule condition, age, and gender can be used to predict contralateral CLNM; capsule condition, age, gender, tumor size, and CLT can be used to predict the number of CLNM. However, how do we choose a surgical procedure based on these predictions? Guidelines on the scope of surgery for IPTCs are lacking (12, 16, 23, 25, 26). Various surgical approaches have been applied to IPTCs, where isthmusectomy, unilateral lobectomy plus isthmusectomy, and total thyroidectomy are the mainstream. The necessity and scope of prophylactic lymph node dissection vary between individuals. For proponents of total thyroidectomy, the aggressive behavior of IPTC makes total thyroidectomy a safer approach compared with partial thyroidectomy (6, 27). Jianyong lei et al. reported that total thyroidectomy has a lower recurrence rate, comparable postoperative complication rate, and faster parathyroid function recovery than partial thyroidectomy (25). For proponents of partial thyroidectomy, isthmusectomy or unilateral lobectomy plus isthmusectomy can avoid postoperative complications and preserve thyroid function. A small cohort of IPTC patients who underwent isthmusectomy, reported by Nixon et al., showed a 100% 10-year disease-specific survival and a 100% local and regional 10-year recurrence-free survival during a median follow-up of 124 months (16). In the debate about prophylactic lymph node dissection, some researchers think that when there is insufficient evidence of lymph node metastasis, avoiding prophylactic dissection can prevent surgical complications, such as postoperative hypocalcemia and nerve injury. In contrast, other researchers think preserving the central lymph node will increase the risk of recurrence. Chad K Sudoko et al. found that ≥4 CLNM were associated with bilateral PTCs (28). Qiang Chen et al. also reported that the total number of prelaryngeal and pretracheal lymph node metastases could be a good factor in predicting contralateral CLNM in patients with unifocal PTC (29). The results of these studies indicated that preservation of the contralateral thyroid is feasible when the number of lymph node metastases is few.

In this study, we constructed prediction models based on risk factors identified by logistic regression, which performed well in the internal validation. Based on the prediction model, we proposed a risk stratification model for lymph node metastasis of IPTC, stratifying patients into three risk groups. We offered different treatment options for each risk group accordingly. For patients with a low risk of ipsilateral CLNM, prophylactic ipsilateral CND should be avoided to reduce surgical complications. Prophylactic CND can be considered for patients with a moderate risk of ipsilateral CLNM. For patients in the high-risk group, prophylactic ipsilateral CND is highly recommended to reduce the chance of recurrence. The individualized treatment decision can also be made in the same way for patients with different risks of contralateral CLNM. For patients with a low risk of contralateral CLNM, prophylactic contralateral CND should be avoided. Prophylactic CND can be considered for patients with a moderate risk of contralateral CLNM. For patients in the high-risk group, prophylactic contralateral CND is highly recommended. Finally, we used the nomogram of the number of CLNM to assess the risk of CLNM. For patients with a low risk of having 1 to 4 CLNM, unilateral lobectomy with isthmusectomy or isthmusectomy alone is recommended. Total thyroidectomy is highly recommended for those with a high risk of 1 to 4 CLNM and a moderate risk of ≥5 CLNM. For patients with a moderate risk of 1 to 4 CLNM and a moderate risk of ≥5 CLNM, unilateral lobectomy with isthmusectomy or isthmusectomy alone should be considered with caution. Our model includes only a few highly accessible variables, making it practical and convenient for clinical practice.

Another noteworthy finding of the study is the association between Hashimoto’s thyroiditis (HT) and CLNM. As we all know, there have been debates about the relationship between HT and differentiated thyroid carcinoma. Some studies identified HT as an independent risk factor for thyroid cancer. The proportion of multifocality in PTC patients with HT is higher than in those without, while the incidence of lymph node metastasis is comparable (30, 31). Salvatore Ulisse et al. proposed autoimmune thyroid disease as a predictive factor for the survival and relapse of PTC (32). However, some studies revealed negative correlations between HT and extrathyroidal extension, lymph node metastasis, distant metastasis, and cancer relapse, demonstrating HT as a protective factor for DTC (33). This study found that HT is a protective factor for CLNM in PTC patients reducing the number of CLNM.

This study has several limitations that should be acknowledged. First, this is a retrospective study, susceptible to inherent biases in patient recruitment and data collection. In order to obtain a sufficient sample size, we prolonged the time span of included medical records, which may cause variance in the number of lymph node dissections. In this case, we standardized the extent of dissection to guarantee the reliability of the study. Second, this study excluded patients with multifocal PTCs which might affect the applicability and accuracy of the model. Finally, although our model showed good predictive power, strict external validation is required in prospective multicenter institutional trials to obtain more objective conclusions. Future studies are needed to refine these shortcomings. We will conduct a prospective cohort study to compare the prognosis of the major surgical procedures for IPTC. In addition, external validation will be conducted to improve the credibility and clinical applicability of the model.

## 5 Conclusion

Here we provided a new prediction tool to predict the location and number of CLNM in patients with IPTC. We proposed a risk stratification schema based on the models and provided corresponding surgical treatment recommendations for patients with IPTC. We hope our study will be helpful for clinicians to provide individualized treatment for patients with IPTC and avoid inappropriate surgical options.

## Data availability statement

The original contributions presented in the study are included in the article/supplementary material. Further inquiries can be directed to the corresponding authors.

## Ethics statement

Ethical review and approval was not required for the study on human participants in accordance with the local legislation and institutional requirements. Written informed consent to participate in this study was provided by the participants’ legal guardian/next of kin.

## Author contributions

YZ and CPL designed the study. YZ, WS, FD, and XW acquired the data., YZ, and WS acquired and analyzed the data. YZ performed the statistical analyses, wrote, and submitted the manuscript. CPL, and CL revised the manuscript. All authors contributed to the article and approved the submitted version.
